# The Diagnostic Challenges and Clinical and Serological Outcome in Patients Hospitalized for Suspected Lyme Neuroborreliosis

**DOI:** 10.3390/microorganisms10071392

**Published:** 2022-07-11

**Authors:** Violeta Briciu, Mirela Flonta, Daniel Leucuța, Mihaela Lupșe

**Affiliations:** 1 Department of Infectious Diseases, “Iuliu Hatieganu” University of Medicine and Pharmacy, 400348 Cluj-Napoca, Romania; mihaela.lupse@yahoo.com; 2 The Clinical Hospital of Infectious Diseases, 400348 Cluj-Napoca, Romania; mflonta4@gmail.com; 3 Department of Medical Informatics and Biostatistics, “Iuliu Hatieganu” University of Medicine and Pharmacy, 400349 Cluj-Napoca, Romania; dleucuta@umfcluj.ro

**Keywords:** antibiotic treatment, *B. burgdorferi* intrathecal antibody index, *Borrelia burgdorferi* sensu lato, clinical outcome, Lyme neuroborreliosis, serological profile

## Abstract

The aim of our study was to evaluate the differential diagnosis and clinical/serological outcome to antibiotic treatment in patients hospitalized for suspected Lyme neuroborreliosis (LNB). A prospective study included patients hospitalized in a Romanian hospital between March 2011 and October 2012 with neurological symptoms, positive laboratory tests for *Borrelia burgdorferi*, cerebrospinal fluid (CSF) analysis, and no previous treatment for LNB. A questionnaire was completed for each patient at admission, at the end of treatment, and 3 months later. Patients were treated with antibiotic therapy (ceftriaxone/cefotaxime), irrespective of CSF analysis results. A symptomatic scoring scale was used for the follow-up. Out of the 42 patients included, no patient fulfilled criteria for definite LNB; 7 patients were classified as possible LNB; and in 33 patients, LNB was excluded. Two patients could not be classified (insufficient amount of CSF). Clinical follow-up suggested a better response to therapy in the group of patients with possible LNB than in the group with LNB excluded. The patients’ differential diagnosis and serological follow-up are presented. Patients investigated for suspected LNB present diverse clinical manifestations and comorbidities that complicate differential diagnosis. LNB may be misdiagnosed if CSF analysis is not performed.

## 1. Introduction

Lyme neuroborreliosis (LNB) represents the manifestation of central and/or peripheral nervous system infection with *Borrelia burgdorferi* sensu lato (s.l.) bacteria that are transmitted through the bite of infected ticks. In Europe, it is caused by *Borrelia garinii* and *Borrelia bavariensis*, less frequently by *Borrelia afzelii*, and rarely by *Borrelia burgdorferi* sensu stricto (s.s.) and *Borrelia bissettiae*. In contrast, in North America, all manifestations of Lyme borreliosis (LB), including LNB, are caused by *B. burgdorferi* s.s. [1]. The clinical manifestations of LNB are diverse and different in American and European patients, probably due to different genospecies. LNB may mimic other neurological diseases and patients suffering from neurological disorders such as multiple sclerosis (MS) and amyotrophic lateral sclerosis (ALS), and polyneuropathy may be misdiagnosed as LNB [2]. The European diagnostic criteria of LNB recommend cerebrospinal fluid (CSF) analysis for intrathecal antibody production detection [3,4,5,6], but compliance with diagnostic lumbar puncture may be cumbersome in clinical practice. Currently, the antibiotic treatment most often used for patients with LNB is a 2-week (for early LNB) or 3-week (for late LNB) course of antibiotic therapy, usually with intravenous ceftriaxone at a dosage of 2 g daily. Most patients with LNB respond well to antimicrobial therapy and objective neurological symptoms, resolving in the vast majority of patients, albeit gradually in some cases. Some patients, however, report subjective symptoms after treatment [1,4]. In this study, we addressed the question if the apparent poor response to antibiotic treatment in our patients is due to an incorrect diagnosis. 

## 2. Materials and Methods

### 2.1. The Study Design

A prospective study included patients hospitalized in the Clinical Hospital of Infectious Diseases, Cluj Napoca, Romania (an academic referral center), with the suspicion of LNB between March 2011 and October 2012. The inclusion criteria were as follows: neurological manifestations, positive laboratory tests for *B. burgdorferi*, lumbar puncture (LP) for diagnosis, and no previous parenteral antibiotic treatment for LNB.

The study was approved by the Ethics Committee of the “Iuliu Hatieganu” University of Medicine and Pharmacy. 

Patients included in the study were previously evaluated clinically and serologically (ELISA and Western blot testing for *B. burgdorferi* serum antibodies in each patient) in our tertiary referral hospital ambulatory. All the patients that presented neurological manifestations and (1) ELISA and Western blot tests positive for *B. burgdorferi* or (2) a negative ELISA test with a positive Western blot test for *B. burgdorferi* were further invited in the study. We excluded patients with positive ELISA and negative Western blot or patients with both tests negative. Each person received information on the aims and the protocol of the study and was included in the study after signing the informed consent form. In the case of persons under the legal age of consent (<18 years old), one of the parents signed the consent form.

All included patients were followed by the principal investigator of the study, an infectious disease specialist. A questionnaire was completed at admission by the principal investigator regarding previous or present co-existing diseases, tick bites, or erythema migrans (EM) history and neurological, musculoskeletal, cutaneous, cardiac, or ocular signs and symptoms. The patients were further evaluated, depending on the clinical symptomatology, in the “Lyme Borreliosis Center”, in a multidisciplinary team (infectious diseases specialist, clinical microbiologist, neurologist, rheumatologist, ophthalmologist, psychiatrist, cardiologist). Cerebral magnetic resonance imaging (MRI) was performed, if indicated by the neurologist for differential diagnosis. A blood sample was collected at the time LP was performed.

Case definition of LNB according to the European guideline [3] used in the study was:

1. Neurological symptoms suggestive of LNB without other obvious reasons.
2. CSF pleocytosis.
3. Intrathecal *B. burgdorferi* antibody production.

Definite LNB: All three criteria fulfilled.

Possible LNB: Two criteria fulfilled.

After the LP was performed, antibiotic treatment was initiated for LNB as recommended [4,5], irrespective of and without knowledge of the CSF analysis results (neither the patient nor the investigator). The antibiotic therapy used was ceftriaxone 2 g/day for 21 days, or cefotaxime 3 × 2 g/day for 21 days in case of patients with cholelithiasis. Gall bladder examination by ultrasonography was performed in all patients before antibiotic therapy was started. The patients were clinically examined daily throughout the therapy. The adverse reactions to medications were noted. Three months post-treatment, the patients were reevaluated clinically and serologically. The questionnaire was repeated at the end of treatment and 3 months post-treatment.

### 2.2. CSF Analysis

LNB is associated with elevated cell count in the CSF, typically 10–1000 leucocytes/mm^3^, with a substantial number of patients having elevated CSF protein. To prove intrathecal production of *B. burgdorferi*-specific antibodies, calculations that consider blood/CSF barrier dysfunctions (IAI) based on quantitative ELISA were used [3]. The CSF cell count, proteinorachia, and glicorachia were determined. The intrathecal antibody index (IAI), using a Genzyme Virotech kit in 2011 and an Euroimmun kit in 2012, was calculated. As recommended by the manufacturer, an IAI < 1.3 shows no production of *Borrelia*-specific antibodies in the CSF, while a value >1.5 shows a local production of specific antibodies and sustains the diagnostic of LNB. A value between 1.3 and 1.5 is considered borderline.

### 2.3. Serological Analysis

Serological testing performed at inclusion used a Genzyme Virotech ELISA kit in 2011/an Euroimmun ELISA kit in 2012 (due to hospital acquisition policy), as well as an Euroimmun Western blot kit. The ELISA test was repeated three months post-treatment. To exclude other infections that may cause cross-reactivity, the patients were tested for HIV infection (VIDAS^®^ HIV DUO Quick-BioMérieux SA, Marcy-l’Étoile, France), *Mycoplasma pneumoniae* infection (Mycoplasma IgM ELISA-Zeus Scientific, Branchburg, NJ, USA), viral hepatitis B (Monolisa™ Hbs Atg ULTRA—Bio-Rad, Marnes-la-Coquette, France), viral hepatitis C (HCV Ab-DIA.PRO, Sesto San Giovanni, Italy), and syphilis (IMMUTREP^®^RPR—Omega Diagnostics, Alva, UK). The presence of the rheumatoid factor was also tested by immunoturbidimetric assay using COBAS C 501 Analyzer (Roche Diagnotics, Basel, Swizerland).

### 2.4. Data Analysis

Continuous normally distributed variables were reported as mean and standard deviation (SD), and categorical variables were presented as frequencies and percentages. In the absence of objective measures for cure after antibiotic therapy in LB patients, we created a symptomatic scale that depicts symptoms’ evolution, presented and used in a previous publication [7]. The patients’ symptoms were followed at admission/at the end of treatment/3 months post-treatment, using the following scale: the presence of a symptom at inclusion and persistance at end of treatment and follow-up = 3 points (pts); symptom decreased in intensity at the end of treatment = 2 pts; symptom decreased in intensity at follow-up compared to end of treatment = 1 pt; absence of the symptom = 0 pts; symptom increased in intensity at the end of treatment = 4 pts; symptom increased in intensity at follow-up compared to the end of treatment = 5 pts.

Two negative binominal mixed effects regression models were used. The dependent variables were the number of symptoms and the symptomatic score, respectively. The explanatory variable, LNB, was coded absent versus possible. The time (corresponding to the observations made), measured in weeks, was used as a random variable. The number of symptoms was defined as the sum of symptoms present in a patient in each of the three moments of observation from the study. The symptomatic score was defined as the sum of points from the symptomatic scores present in a patient in each of the three moments of observation from the study.

The statistical analysis used R environment for statistical computing and graphics, version 2.15.1 (R Development Core Team, 2012) [8].

Categorical variables were compared using chi-square tests, or if expected cell frequencies were small, Fisher’s exact test was used. For all tests, a level of significance of 0.05 was chosen, and the two-tailed *p*-value was used. Quantitative data not following the normal distribution were presented using box plots. Bar charts were used to describe qualitative data.

## 3. Results

### 3.1. Patient Characteristics at Inclusion

A total of 42 patients were included, 17 patients in 2011 and 25 patients in 2012. Patients’ demographic data are presented in Table 1.

### 3.2. Patient Diagnosis Classification

The CSF analysis showed proteinorachia (more than 40 mg/dL) present in 20 patients, mean ± SD= 40 ± 14.6 mg/dL (min: max = 18.9–102.5 mg/dL). Pleocytosis was present in four patients: 6, 10, 21, and 58 leucocytes/mm^3^ respectively (Table 2).

Fifteen patients presented *Borrelia*-specific antibodies in CSF, but the IAI was positive in three patients (one patient with positive IgM IAI, two patients with positive IgG IAI) and negative in nine patients, while for three patients, it could not be calculated due to the insufficient amount of CSF obtained during the LP—no CSF left for determining total IgG in CSF (Table 2). Nevertheless, one of the three patients had pleocytosis and was classified as possible LNB. Twenty-seven patients did not present *Borrelia*-specific antibodies in the CSF.

On the basis of these results, no patient fulfilled criteria for definite LNB; 7 patients were classified as possible LNB; and in 33 patients, LNB was excluded. Two patients could not be classified.

### 3.3. Clinical Data, Serology Results, and Imaging—Associations with Possible LNB Diagnosis

We looked for associations between the diagnostic of possible LNB and tick bite history, previous EM lesions, symptoms and signs at inclusion, serological tests at inclusion, and demyelinating lesions on cerebral MRI (Table 3).

No association was proven between the history of tick bites or previous EM lesions and the diagnosis of possible LNB (*p* = 0.0948 and *p* = 0.204, respectively).

We did not identify symptoms/signs that appear more frequently (statistically significant) in the group of patients with possible LNB than in the group with LNB invalidated. We identified differences between the two groups only regarding paresthesia, more frequently present at inclusion in the group with LNB invalidated. Apart from the neurological symptoms, our patients presented musculoskeletal or general symptoms (see the Supplementary Materials).

Twenty-nine patients (69%) presented both ELISA and Western blot tests positive at inclusion, while the rest of the 13 patients presented a negative ELISA test with a positive Western blot test (Supplementary Materials). We evaluated the association between serological test at inclusion (positive ELISA + positive Western blot or negative ELISA + positive Western blot) and the diagnosis of possible/invalidated LNB. No association was proven (*p* = 0.39).

The cerebral MRI with T1-weighted, T2-weighted, fluid-attenuated inversion recovery (FLAIR), and diffusion-weighted sequences (plus contrast enhancement if recommended by the radiologist) was performed in 37 patients. No association was proven between the presence of demyelinating lesions on cerebral MRI and the diagnostic of possible/invalidated LNB (*p* = 1).

The exhaustive list of patients’ symptoms and signs present at inclusion and followed by the symptomatic scale during the study are presented in the Supplementary Materials.

### 3.4. Differential Diagnosis

All the patients were evaluated by a multi-specialty medical team regarding differential diagnosis and/or comorbidities. In 22 patients, the neurologist established, on the basis of clinical and paraclinical neurological examination, the following diagnoses, apart from suspected LNB, as presented in Table 4.

For these patients, specific therapy recommended by the neurologist for the established diagnoses was initiated immediately or followed the antibiotic therapy.

Although the remaining 20 patients included in the study complained of diverse nonspecific neurological symptoms, the neurologist, on the basis of the clinical and paraclinical neurological examination, could not establish other neurological diagnoses apart from suspected LNB.

A rheumatological diagnosis was established by the rheumatologist in nine of the patients included in the study; diagnoses are presented in the Supplementary Materials.

### 3.5. Therapy

In our study group, 39 patients were treated with ceftriaxone and two with cefotaxime. One patient started treatment with ceftriaxone but after five days presented vomiting and abdominal pain; biliary sludge was documented by abdominal ultrasonography (although it was not present before therapy) and treatment was changed to cefotaxime, well tolerated. Thirteen patients (30.9%) presented multiple adverse reactions to medication (such as diarrhea, *Clostridium difficile* infection, vomiting, nausea, abdominal pain, aphtous stomatitis, vaginal candidiasis, skin rash).

Antibiotics were stopped in two cases: skin rash (11th day of therapy) and *Clostridium difficile* infection (14th day of therapy). The rest of the adverse reactions were treated with specific therapy while antibiotic therapy was continued.

The specific therapy for the neurological and/or rheumatological condition was associated or followed the antibiotic therapy, as recommended by the specialist (anti-inflammatory therapy, nootropic therapy, therapy for neuropathic pain or specific psychiatric medication).

### 3.6. Clinical Follow-Up

In the absence of objective measures for cure after antibiotic therapy in our study group patients, the clinical outcome was defined as the evolution of the number of symptoms or symptomatic score from the inclusion through the follow-up.

Thirty-six patients presented at follow-up out of the 42 patients included in the study: all the seven patients classified as possible LNB, 27 of the 33 patients with LNB invalidated, and the two patients not classified.

**Figure 1:**
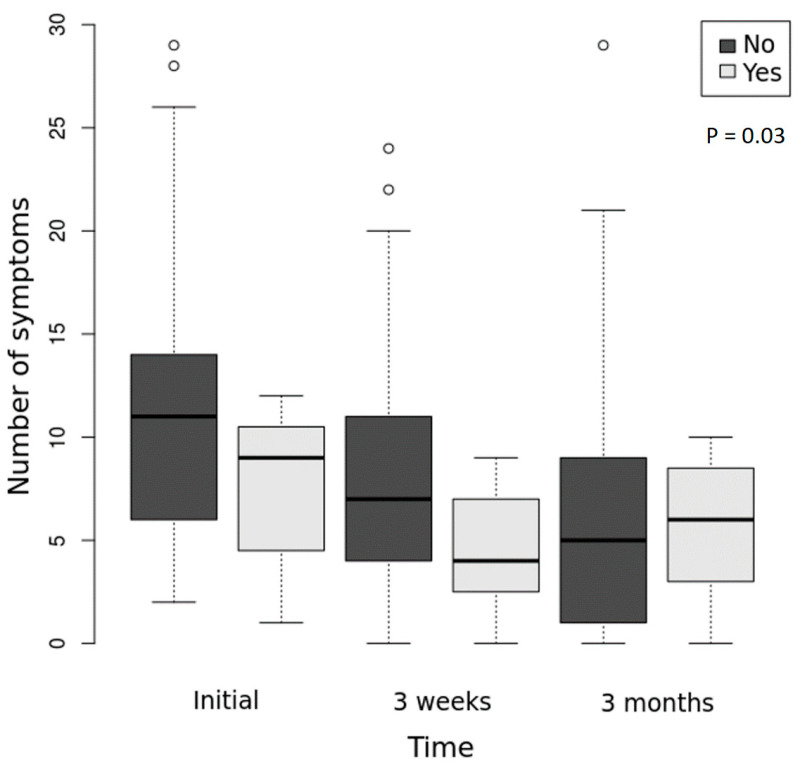
Evolution of the number of symptoms in the two groups (possible LNB versus invalidated LNB) during the follow-up. Box represents median observations (horizontal rule) with 25th and 75th percentiles of observed data (top and bottom of box). The length of each whisker is 1.5 times the interquartile range (Yes = possible LNB; No = LNB invalidated). The overall differences presented as *p*-value on the chart, between the two groups, were assessed with a negative binominal mixed effects regression model.

Figure 1 presents the evolution of the number of symptoms in the two groups of patients.

The spectrum is narrower in the group of patients with possible LNB.

Results of the regression models showed a better clinical evolution in the group of patients with possible LNB than in the group with LNB invalidated; patients with LNB invalidated had more symptoms through the follow-up, statistically significant (exponentiated coefficients: 1.530, *p* = 0.03), than those with possible LNB, as well as a higher symptomatic score, close to the limit of statistical significance (exponentiated coefficients: 1.502, *p* = 0.055).

During the follow-up, two patients from the invalidated LNB group were confirmed as ALS, and one patient was confirmed as MS (Table 4). Two of these patients presented a negative ELISA test (with positive IgG and borderline IgG, respectively, on Western blot), while the third case presented positive IgM both on ELISA and Western blot, although the clinical symptomatology was present since 2001.

### 3.7. Serological Follow-Up

**Figure 2:**
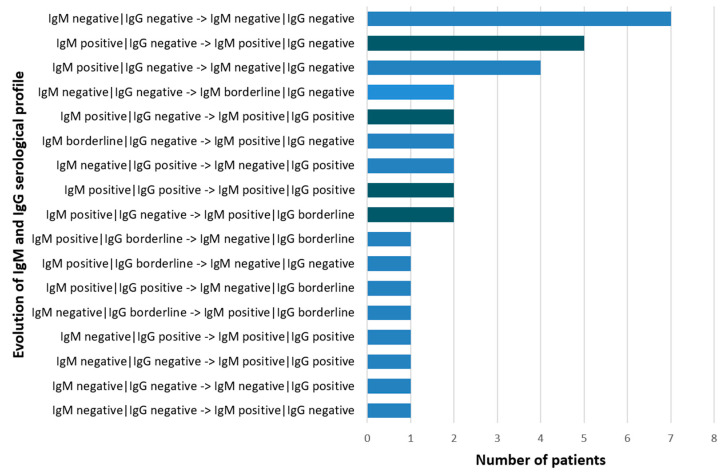
Serological profile of IgM and IgG anti-*B. burgdorferi antibodies*: IgM/IgG at inclusion –> IgM/IgG at follow-up. The symbol arrow “–>” means evolution from inclusion to follow-up. Persistent IgM positive profiles are represented as bars with darker color.

A total of 36 out of the 42 patients included in the study presented for the follow-up. We evaluated the serological profile at inclusion and 3 months post-treatment with regard to distinct profiles of kinetics for each antibody class (Figure 2).

The results underline patients’ diverse serological profiles throughout the follow-up. As both ELISA tests used in our study presented sensitivities of >99% (according to the manufacturers), the use of the two different tests had no influence on the diverse serological profile of the investigated patients. No case of seroconversion (IgM positive/IgG negative–IgM negative/IgG positive) as an argument for acute *B. burgdorferi* infection was described. Eleven patients presented persistent IgM positive profile (columns with darker color in Figure 2); in five of them, this was associated with a persistent IgG negative profile.

In two patients, one classified as possible LNB and one as invalidated LNB, the serological reaction for *Mycoplasma pneumoniae* (IgM) was positive. As cross-reactivity represents a limit in the serological diagnosis of LB, doxycycline, recommended for therapy of *Mycoplasma pneumoniae* infection, followed the therapy with ceftriaxone. All the serological tests performed for HIV, hepatitis B and C infections, and syphilis were negative.

## 4. Discussion

In the presence of positive serology for *B. burgdorferi* s.l. in the asymptomatic population [9,10,11], the development of neurological symptoms suggestive of LNB may pose diagnostic challenges. A causative relation between exposure to *B. burgdorferi* and different neurological disorders was suspected, but seroprevalence studies did not confirm this hypothesis, as reported prevalence in patients with peripheral polyneuropathy [10], MS [12], Alzheimer’s disease [13], and ALS [14] was similar or lower than prevalence found in the healthy population in the same geographical areas. The risk of false-positive LNB diagnosis is high if CSF criteria are not fulfilled and the diagnosis is based on neurological symptomatology and positive specific serum antibodies (as compliance with LP can be cumbersome in clinical practice). None of the 42 patients in our study fulfilled the criteria for definite LNB, and only 16.6% of the cases were classified as possible LNB. Almost one-third of the patients (11 out of 36 patients that were assessed at the 3 months’ follow-up) presented persistent positive IgM serological profile, suggesting possible false-positive reaction to *B. burgdorferi*. False-positive reactions have been described in patients with autoimmune diseases such as MS, ALS, SLE, or thyroiditis [15,16,17,18]. In our study, a patient was confirmed as MS and two others as ALS during the 3 months follow-up. Two of these patients presented a negative ELISA screening test with positive Western blot test. The use of immunoblot in a single step is currently not recommended as it increases the risk of false-positive results by reducing test specificity [19]. The third case presented both ELISA and Western blot as positive for IgM antibodies, although the clinical symptomatology was present for several years, interpreted as persistent or unspecific IgM that argues against a long (chronic) course of LNB. To improve the accuracy of serologic testing for LB, it has been recommended to limit IgM immunoblot testing to patients with symptoms and signs of recent onset [18]. Although diagnostic criteria for MS have been established [20], LNB is frequently indistinguishable from MS on clinical and radiologic grounds. White matter lesions similar to those present in MS have been reported on T2 and FLAIR MR imaging scans of patients with LNB [21,22,23,24]. In our study, no association could be proven between the presence of cerebral demyelinating lesions and the diagnosis of possible LNB. Agarwal and Sze (2009) [24] showed that MR imaging findings of the brain are rare in LNB, and white matter lesions may be incidental in LNB patients, concluding that LNB should not be considered crucial in the differential diagnosis of foci of T2 prolongation in the cerebral white matter, particularly in middle-aged and elderly patients.

None of the three patients diagnosed during the follow-up with MS and ALS presented improvement after antibiotic therapy. A study that has evaluated the incidence of *B. burgdorferi* antibodies in 283 patients with MS reported positive or borderline results in 19 of them, with no clinical benefits after antibiotic therapy [15]. A Norwegian study that evaluated 122 patients with unspecific neurologic symptoms and positive serology for *Borrelia* diagnosed MS in 13 of the patients [25]. The prescription of antibiotics with no definite diagnosis is no exception in clinical practice, being also described in other countries endemic for *B. burgdorferi*. A study performed in USA evaluated the consequences of overdiagnosis and overtreatment of LNB, showing that only 3% of the patients treated were fulfilling the CDC diagnostic criteria [26]. In another therapeutic study [27], only 21% of the treated patients fulfilled the diagnostic criteria. Adverse reactions to medication, nosocomial infections and bacterial resistance to antibiotics are problems that may follow the overuse of antibiotics in patients with not confirmed LB. The patient with *Clostridium difficile*-associated infection included in our study had a favorable outcome under specific therapy, but death caused by this infection after prolonged antibiotic treatment for LB has been reported [28].

The evaluation of response to treatment in patients with LB is difficult in the absence of objective measures for cure. The persistence of symptomatology after antibiotic therapy is a phenomenon much disputed and studied. The frequency and characteristics of these symptoms vary [29], and the explanation for their persistence is not clear, in spite of numerous studies including clinical trials [30,31,32,33], studies on reactivation of atypical spirochetal forms [34], or the study of psychiatric comorbidity [35]. The persistence of *B. burgdorferi* after antibiotic therapy has been documented on animal models [36,37,38], but ticks could not transmit the infection from the infected mice to other mice. It was suggested that the persistent spirochetes were noninfectious and attenuated, or they represented a subpopulation of “persisters” tolerant to antibiotics. Ljøstad and Mygland (2010) [39] have shown that in the case of early LNB, the symptoms persist after treatment in 48% of the patients, and the identified risk factors were greater than six weeks duration of symptomatology before treatment, high pleocytosis, and female gender. The absence of definite LNB in our study group and the small number of cases did not allow us to evaluate the risk factors for unfavorable outcome. In the group of patients diagnosed with possible LNB, articular manifestations were described, possibly due to *B. burgdorferi* infection (scapulo-humeral periarthritis, reactive arthritis) but also to comorbidities (i.e. Haglund disease). These musculoskeletal manifestations that have a slow response to antibiotic and anti-inflammatory therapy may explain some of the remaining complaints 3 months post-treatment. For the two patients diagnosed with possible LNB and arthritis, due to persistent articular complaints, a second oral regimen with doxycycline was prescribed, according to guidelines in use [4,40].

Our study has the limitation of including in the study group the 13 patients with negative ELISA tests and positive Western blot test. We addressed these patients with the suspicion of LNB for further CSF investigation based on clinical decision, as it is known that in some cases, antibodies are detectable in CSF whilst negative in serum, and diagnostic sensitivity of ELISA screening assays in early LNB is 70–90% [3,5]. The only patient from this subgroup further classified as possible LNB on the basis of a positive IAI showed seroconversion at 3 months follow-up with the presence of IgG antibodies in serum on the ELISA test (Supplementary Materials). The neurological diagnosis of the patient was right peripheral vestibular disorder and vascular encephalopathy (Table 4). These data support the idea of the limitation of serum tests in patients with early neurological manifestations and indication, on the basis of clinical decision and multidisciplinary approach, for further CSF investigation. This is in accordance with the case definition of LNB according to European guidelines that do not include positive two-tier testing in serum for definite LNB, nor LP for diagnostic only in two-tier-positive patients, as the diagnostic in clinically suspected LNB cases is based on CSF investigation as the first diagnostic tool [1,3,5,6].

Although the most recent European guidelines [1,6] recommend PCR or CXCL13 testing in CSF in patients with negative IAI when LNB is still suspected, these recommendations were not included in the guideline available for case definition in our study group [3], as presented in the Materials and Methods section. By the time the study was performed, there was not enough evidence to recommend CXCL13 test as a routine diagnostic tool, and PCR in CSF was recommended in very early LNB with negative IAI, or in patients with immunodeficiency, on the basis of good practice points [3].

In our study group, none of the patients investigated fulfilled the three criteria for definite LNB. The results suggest a better outcome in the group of patients with possible LNB than in the group of patients with LNB invalidated, as patients with LNB invalidated had more symptoms through the follow-up; were statistically significant; and had a higher symptomatic score, close to the limit of statistical significance, than those with possible LNB. These findings support the statement of Puéchal and Sibilia (2009) [41] that the most common cause of treatment failure is an incorrect diagnosis and patients should be thoroughly examined for medical conditions that could explain the symptoms. Patients with persisting neurological symptoms but no CSF changes should be reevaluated for medical conditions that benefit from a specific therapy.

Misdiagnosis and underdiagnosis are the two medical errors that clinicians may face regarding LB. In our region, where ticks are infected with the main *B. burgdorferi* human pathogenic species [11,42], underdiagnosis might be caused by not investigating patients with neurological symptomatology for LNB, while misdiagnosis might be caused by lack of differential diagnosis in symptomatic patients with positive serology for *B. burgdorferi*. Because *B. burgdorferi*-specific antibodies may persist 10–20 years after infection [43] and seroprevalence in the general population is high in our region [11], comorbidities, physiological aging, stress, and publicity on LB should be considered when investigating patients that present with the suspicion of LB. A good interdisciplinary collaboration represents the key in the diagnosis and management of these patients. The differential diagnosis should be evaluated in the multidisciplinary team [4]. To provide better care for patients suspected of having LB, we founded in 2010 the “Lyme Borreliosis Center”, with a multidisciplinary team (infectious diseases specialist, clinical microbiologist, neurologist, rheumatologist, ophthalmologist, psychiatrist, cardiologist) similar to centers reported by other academic referral centers [44,45,46,47]. All the patients included in our study were evaluated from that perspective, with no difference if they were classified as possible or invalidated LNB. As we have also underlined in our study, on a smaller number of patients addressed to our academic referral center with suspected LNB, up to 85% of more than 1000 patients included in three French studies for suspected LB actually received another diagnosis, with a potential loss of chance for appropriate care because of diagnostic delay, and up to 85% of patients received a pointless antibiotic therapy (sometimes for years) [45,46,47]. As in our study, the previous mentioned studies confirmed the wide range of differential diagnoses: neurological diseases, rheumatologic diseases, psychiatric or psychological diseases, or systemic/autoimmune diseases. Because medical information regarding LB available through the internet and mass media may be contradictory [48], our patients have been informed about the risks of prolonged antibiotic therapy, with unproven benefits and possible adverse reactions [4].

Regarding national epidemiological data on LB in Romania, over the past 10 years, the incidence of LB has varied between 1.2 and 4.2 cases per 100,000 individuals annually, with an incidence rate in 2011 (the year when our study was initiated) of 2 cases/100,000 inhabitants [49]. In a previous publication, we presented a case series of 44 EM patients diagnosed in 2011 in our hospital [50]. In the same year, surprisingly at a national level, only one LB case was reported from our county [51]. These data argue that the real number of LB patients in Romania may be widely underreported. The majority of reported cases in 2018, the last year published by the Romanian National Institute of Health, were represented by early LB (90%), mainly EM, with only five cases of LNB. We found only one case series study on LNB published from one of Romania’s counties, including 50 patients in three years [52].

LNB was included in 2018 on the list of diseases under EU epidemiological surveillance, with a uniform EU case definition being formally released [53]. Diagnostic difficulties, under- and overreporting, and different laboratory methods used remain important issues for LNB diagnosis and surveillance in Europe [54]. Although ECDC will start monitoring LNB distribution through the epidemiological surveillance network, until the diagnosis and reporting system is improved at the national level, the real incidence of LNB in all European countries will remain unknown.

## 5. Conclusions

Patients investigated for suspected LNB present diverse clinical manifestations and comorbidities that complicate differential diagnosis. LNB is misdiagnosed if the European definition criteria is not followed. Our study is strengthening the low frequency of LNB diagnoses in patients consulted for presumed LNB even in an academic referral center. Considering the variety of differential diagnosis in patients investigated for suspected LNB, a multidisciplinary management with different specialists should be considered in all reference centers.

## Figures and Tables

**Table 1:** Demographic characteristics of the studied patients.

Characteristics	
Number of adults: children	41:1
Age (years): mean ± SD (min-max)	35.83 ± 13.85 (4–63)
Female: male, number (%)	33 (78.57): 9 (21.43)
Urban: rural residence, number (%)	34 (80.9): 8 (9.1)

SD = standard deviation.

**Table 2:** LNB classification according to CSF analyses of pleocytosis and intrathecal *B. burgdorferi* antibody production.

	Possible LNB		Invalidated LNB		Not Classified LNB	Total
	Pleocytosis	No Pleocytosis	Pleocytosis	No Pleocytosis	No Pleocytosis	
CSF antibodies						15
VIAI > 1.5	0	3	0	0	0	3
IAI < 1.3	0	0	0	9	0	9
IAI not determined	1	0	0	0	2	3
No CSF antibodies	3	0	0	24	0	27
Total	4	3	0	33	2	42

LNB = Lyme neuroborreliosis; IAI = intrathecal antibody index; CSF = cerebrospinal fluid. Data represent number of subjects.

**Table 3:** Clinical data, serology results, and cerebral magnetic resonance imaging associations with possible LNB diagnosis.

Characteristics	Total	Possible LNB n (%)	LNB Invalidated n (%)	*p*-Value
Tick bite recalled	19	1 (14.3)	18 (54.5)	0.095
Erythema migrans	5	2 (28.6)	3 (9.1)	0.204
Signs and symptoms				
Cervical pain	11	0 (0)	11 (33.33)	0.159
Decrease in occupational activity	16	3 (42.86)	13 (39.39)	1
Decrease in visual acuity	9	1 (14.29)	8 (24.24)	1
Diplopia	5	0 (0)	5 (15.15)	0.565
Facial paresis	2	0 (0)	2 (6.06)	1
Fatigue	27	4 (57.14)	23 (69.7)	0.662
Gait disorders	14	0 (0)	14 (42.42)	0.075
Headache	23	5 (71.43)	18 (54.55)	0.677
Joint pain	21	2 (28.57)	19 (57.58)	0.226
Joint tumefaction	3	0 (0)	3 (9.09)	1
Memory impairment	13	1 (14.29)	12 (36.36)	0.393
Myalgia	18	5 (71.43)	13 (39.39)	0.211
Optic neuropathy	2	0 (0)	2 (6.06)	1
Paresthesia	32	3 (42.86)	29 (87.88)	0.02
Photophobia	6	1 (14.29)	5 (15.15)	1
Speech disorders	15	2 (28.57)	13 (39.39)	0.691
Tremor	12	1 (14.29)	11 (33.33)	0.652
Vertigo	19	3 (42.86)	16 (48.48)	1
Serology				
Negative ELISA + positive WB	13	1 (14.3)	12 (36.4)	0.393
Positive ELISA + positive WB	27	6 (85.7)	21 (63.6)	
Demyelinating lesions on cerebral MRI	20	4 (57.1)	16 (57.1)	1

LNB = Lyme neuroborreliosis; ELISA = enzyme-linked immunosorbent assay; WB = Western blot; MRI = magnetic resonance imaging.

**Table 4:** The neurological diagnosis in 22 of the studied patients.

	**Neurological Diagnosis**
Possible LNB	Right peripheral vestibular disorder. Vascular encephalopathy.
	Demyelinating cerebral lesions of unknown etiology.
	Acute encephalitis, right hemiparesis, expressive aphasia.
LNB invalidated	Incomplete thoracic myelitis with left hemicorporeal paresthesia syndrome and sensory level at the sixth dorsal segment.
	Tension-type headache, lumbar discopathy with radiculalgia.
	Vertebrobasilar stroke, right hemiparesis, transient ischemic attack.
	Demyelinating disease, persistent headache, severe hypotonia in the lower limbs.
	Demyelinating disease.
	Demyelinating disease.
	Suspicion of MS, progressive bulbar palsy- confirmed during follow-up as ALS.
	Cephalalgia.
	Central vestibular disorder.
	Left sacral 1 radiculopathy.
	Fibromyalgia.
	Axonal peripheral polyneuropathy.
	Right brachial plexus paresis, right Cervical 7 radiculopathy.
	Migraine without aura.
	Suspected ALS-confirmed during follow-up as ALS.
	Left peripheral facial palsy.
	Suspected MS-confirmed during follow-up as MS.
	Suspected MS, left peripheral facial palsy.
	Benign intracranial hypertension, right hemiparesis, anxiety disorder with somatization.

LNB = Lyme neuroborreliosis; MS = multiple sclerosis; ALS = amyothrophic lateral sclerosis.

## Data Availability

Data is contained within the article or Supplementary Materials.

## Supplementary Materials

The following supporting information can be downloaded at: https://www.mdpi.com/article/10.3390/microorganisms10071392/s1, LNB questionnaire & Supplementary data.
