# Supplementary material for: The Diagnostic Challenges and Clinical and Serological Outcome in Patients Hospitalized for Suspected Lyme Neuroborreliosis
Source: Microorganisms. 2022 Jul 11;10(7):1392. doi: 10.3390/microorganisms10071392 (PMC9324737; doi:10.3390/microorganisms10071392)
Supplement: Supplementary file 1 [file microorganisms-10-01392-s001.zip › LNB questionnaire.pdf]

Date:________________________________

Nr.of file:__________________________________

QUESTIONNAIRE FOR LYME BORRELIOSIS

Name and surname ....

Personal identification number .... Date of birth ....

Occupation………………………………………………………………..

Residence:..

Phone number................................

DIAGNOSTIC..
...

Drug allergies (specified)…..
Medical history……….

**Infection risk assessment**	**YES**	**NO**
Animals in the household		
Ticks on animals in the household		
Outdoor recreational activities	**YES**	**NO**
Hunting		
Hikes		
Fishing		
Other (specified)		

**GENERAL SIGNS AND SYMPTOMS**				
	**PRESENCE**			**APPEARANCE** (interval from tick bite)
Tick bite<br>Date……………..<br>Location…………..<br>Number of ticks……	Start of treatment	End of treatment	3 month follow-up	
Red spot (A) / with extension (B) (after tick bite)				
"Summer flue" (fever, chills, rhinorrhea)				
Fatigue (A) / night sweats (B)				
Decline in the work capacity				
Fluctuation of symptoms				
Lymphadenopathy				
Other				
**DERMATOLOGICAL SIGNS AND SYMPTOMS**				
Hardening (A)/ coloring (B) of the skin				
Thinning (A) / hipersnsitivity of the skin (B)				
Nail changes (thin, breaking)				
Alopecia				
Hives				

Other				
**MUSCULOSKELETAL SIGNS AND SYMPTOMS**				
Cervical (A) / dorsal (B) lumbar (C) spine pain				
Muscle hyper (A) / hypotonia (B)				
Pain (A), swelling (B), erythema (C), stiffness (D) of the joints				
Plantar pain				
myalgia				
Arthritis				
Tendinitis				
Synovitis				
Other				
**NEUROLOGICAL SIGNS AND SYMPTOMS**				
Facial paresis				
Vertigo (A), hearing impairment (B)				
Chewing difficulties (A) / swallowing (B)				
Taste changes (A) / olfactory changes (B)				
Language disorders				
Headache (A), migraine (B)				
Paresthesias (A), anesthesia (B), pain (C)				
Cranial nerve involvement				
Guillain-Barre syndrome				
Meningitis				
Strength loss (A), muscle mass loss (B)				
Ataxia (A) / Walking difficulties (B)				
Tremor				
Amnestic cognitive impairemet (short duration (A) / long duration (B))				
Affective disorders: anxiety (A),				
depression (B), panic (C), irritability (D)				
Emotional lability				
Decreased school performance				
Concentration difficulties				
Sleeping disorders				
Nightmares				
Dizziness				
Dementia				
Coordination deficits				
Enuresis				
Cerebral atrophy (imaging)				
Demyelinating lesions or MS appearance (imaging)				
**OPHTALMOLOGICAL SIGNS AND SYMPTOMS**				
Diplopia (A), decreased visual acuity (B)				
Conjunctivitis				
Choroiditis				
Foreign body sensation				
Ocular pain				
Nystagmus				
Photophobia				
Optic neuropathy				

Retinal vasculitis				
Periorbital edema				
Uveitis				
Posterior scleritis				
Other				
**OTHER ORGANS SIGNS AND SYMPTOMS**				
Cardiac rhythm disorders				
Myocarditis (A), pericarditis (B)				
Cardiomegaly				
Ventricular dysfunction				
Precordial pain				
Hepatic impairement				
Abdominal pain				
Anorexia				
Weight loss (A) / weight gain (B)				
Diarrhea				
Vomiting				
Difficulty urinating				
Disorders of sexual dynamics				
Testicular pain				

Time from therapy initiation to:
a. improvement of the first symptom or objective sign …….
b. disappearance of the first symptom or objective sign ……

Aggravation of the symptoms present at admission:
a. in the first 5 days …….
b. after 5 days of therapy ……

Antibiotics side-effects (mention duration in days)
Allergoderma ……
Gastrointestinal manifestations ……
Nausea ……
Vomiting…….
Abdominal pain ……
Diarrhea……
Decreased appetite
Other ……

Antibiotic therapy for other infectious diseases during the 3 months follow-up….

The doctor who informed the patient ……………
